# Ploidy levels in diverse picocyanobacteria from the Baltic Sea

**DOI:** 10.1111/1758-2229.70005

**Published:** 2024-09-17

**Authors:** Julia Weissenbach, Anabella Aguilera, Laura Bas Conn, Jarone Pinhassi, Catherine Legrand, Hanna Farnelid

**Affiliations:** ^1^ Department of Biology and Environmental Science, Centre for Ecology and Evolution in Microbial Model Systems (EEMiS) Linnaeus University Kalmar Sweden; ^2^ Department of Aquatic Sciences and Assessment Swedish University of Agricultural Sciences Uppsala Sweden; ^3^ School of Business, Innovation and Sustainability Halmstad University Halmstad Sweden

## Abstract

In nature, the number of genome or chromosome copies within cells (ploidy) can vary between species and environmental conditions, potentially influencing how organisms adapt to changing environments. Although ploidy levels cannot be easily determined by standard genome sequencing, understanding ploidy is crucial for the quantitative interpretation of molecular data. Cyanobacteria are known to contain haploid, oligoploid, and polyploid species. The smallest cyanobacteria, picocyanobacteria (less than 2 μm in diameter), have a widespread distribution ranging from marine to freshwater environments, contributing significantly to global primary production. In this study, we determined the ploidy level of genetically and physiologically diverse brackish picocyanobacteria isolated from the Baltic Sea using a qPCR assay targeting the rbcL gene. The strains contained one to four genome copies per cell. The ploidy level was not linked with phylogeny based on the identity of the 16S rRNA gene. The variation of ploidy among the brackish strains was lower compared to what has been reported for freshwater strains and was more similar to what has been reported for marine strains. The potential ecological advantage of polyploidy among picocyanobacteria has yet to be described. Our study highlights the importance of considering ploidy to interpret the abundance and adaptation of brackish picocyanobacteria.

## Graphical abstract

The study on ploidy in brackish picocyanobacteria reveals minimal variation in genome copy numbers. Most strains are haploid, with a few oligoploid strains (two to four genome copies). The ploidy levels in brackish strains are lower compared to freshwater strains and more similar to marine strains.

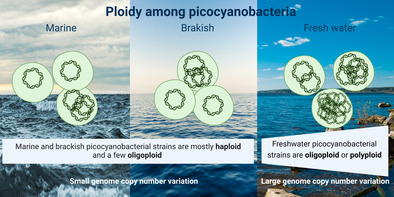

## INTRODUCTION

Ploidy, the number of genome copies within one cell/chromosome, is a well‐known phenomenon in Eukarya and can be found among organisms ranking from ciliates to humans (Comai, 2005; Thorpe et al., 2007; Wendel, 2000). For a long time, prokaryotes were assumed to be haploid (to contain only one circular genome per cell). However, it is now established that oligoploidy (multiple genome copies per cell) and polyploidy (≥10 genome copies per cell) are common among the Bacteria and Archaea domains (Pecoraro et al., 2011; Soppa, 2014; Suh et al., 2001; Tobiason & Seifert, 2006). In recent years, the number of studies sampling environmental DNA and using molecular tools (omics) to characterize microbial communities has increased drastically (Farrant et al., 2016, Sunagawa et al., 2015; Tully et al., 2018). In such studies, the relative abundances of organisms are often estimated based on the assumption that there is only one genome copy per organism. Gaining information about the variation in genome copy numbers in specific organism groups and factors that influence ploidy is therefore of high importance for accurate community composition studies.

Cyanobacteria, photosynthetic prokaryotes, are found in almost all environments. Depending on the geographical location and season, they are represented by different morphological forms ranging from single‐cell to multicellular filamentous species. Picocyanobacteria (<2 μm in diameter) play a significant role in primary production and carbon cycling and have a cosmopolitan distribution in marine, estuarine/brackish, and freshwater ecosystems (Flombaum et al., 2013; Li, 1994; Partensky et al., 1999; Visintini et al., 2021). While the picocyanobacterium *Prochlorococcus* inhabits marine environments, the picocyanobacterium *Synechococcus* has a wider distribution extending from marine to freshwater systems (e.g., Biller et al., 2015; Zwirglmaier et al., 2008). Different strains of picocyanobacteria are adapted to diverse light, temperature, salinity, and nutrient conditions which determine their distribution patterns (Johnson et al., 2006; Laber et al., 2022; Sohm et al., 2016; Xia et al., 2023). In recent years, estuarine and brackish picocyanobacteria have been increasingly recognized (Camacho et al., 2003; Celepli et al., 2017; Sánchez‐Baracaldo et al., 2008) and co‐occurring brackish strains have shown a remarkable variation in ecophysiological adaptations to salinity gradients and light availability (Aguilera et al., 2023; Zufia et al., 2022). It has also been shown that genomic traits, genome size, GC content, and percentage of core genes differ between marine, brackish, and freshwater picocyanobacteria suggesting that they have genetically diversified into defined ecotypes (Cabello‐Yeves et al., 2022).

Picocyanobacteria as a group contain haploid, oligoploid, and polyploid species (Griese et al., 2011). In cyanobacteria, polyploidy has been associated with cell volume, as genome copy number is reported to be proportional to cell size (Ohbayashi et al., 2019; Watanabe, 2020). Potential benefits of polyploidy include improved chromosome repair, resistance to stress conditions, and reduced phage susceptibility (Domain et al., 2004; Watanabe, 2020; Zborowsky & Lindell, 2019). The ploidy level of cyanobacteria can be influenced by the growth phase, growth rate, and the surrounding light and nutrient conditions (Ohbayashi et al., 2019; Riaz et al., 2021; Zerulla et al., 2016). For example, marine *Synechococcus* strains present varying genome copy numbers under different growth conditions such as temperature and exposure to darkness, which affects the cell cycles and therefore, DNA replication (Armbrust et al., 1989; Binder & Chisholm, 1995; Liu et al., 1998; Perez‐Sepulveda et al., 2018). However, the factors that determine ploidy in picocyanobacteria and how it varies among ecotypes are not well understood.

Studies of ploidy level in picocyanobacteria have mainly focused on model strains from freshwater and marine environments (e.g., Griese et al., 2011; Perez‐Sepulveda et al., 2018; Zborowsky & Lindell, 2019) but information on ploidy level of brackish strains is still lacking. Freshwater picocyanobacteria can be oligoploid, containing between two and six genome copies, while several *Synechocystis* sp. PCC 6803 substrains are even classified as polyploid with >10 genome copies depending on the cell cycle (Griese et al., 2011; Zerulla et al., 2016). In contrast, the majority of marine *Synechococcus* and *Prochlorococcus* are haploid with a few oligoploid exceptions (Armbrust et al., 1989; Binder & Chisholm, 1995; Perez‐Sepulveda et al., 2018; Zborowsky & Lindell, 2019). In this study, we investigated the genome copy number of 18 brackish picocyanobacteria from the Kalmar Algae Collection (KAC), recently isolated from the Baltic Sea Proper (7 PSU) (Aguilera et al., 2023). The Baltic Sea, one of the largest brackish water bodies on Earth, is characterized by high nutrient and DOC concentrations, and seasonal temperature variations of >15°C (Bunse et al., 2019). Baltic Sea picocyanobacteria represent a wide physiological and genetic diversity (Aguilera et al., 2023) but brackish picocyanobacteria in general remain understudied compared to their freshwater and marine counterparts. Information on genome copy numbers of brackish strains leads to a better understanding of polyploidy among picocyanobacteria and aids in the interpretation of increasing amounts of omics data.

## EXPERIMENTAL PROCEDURES

### Sample collection and culture conditions

Brackish strains used in this study represent a variety *of Synechococcus* ecotypes from the Baltic Sea Proper (coastal station in the Kalmar Sound 56°39′24.4″N, 16°21′36.6″ E) and Linnaeus Microbial Observatory (LMO 56°55′512.4″N, 17°3′38.52″ E). The brackish strains were obtained from the KAC. Intense ecophysiological characterization of the selected strains has been recently done (see details in Aguilera et al., 2023). *Synechococcus* strains WH8102 and WH7803 were purchased from the Roscoff Culture Collection (RCC; Roscoff, France), Cultures were grown in L1 media prepared with artificial seawater (7 PSU for brackish strains, 33 PSU for marine strains) grown at 16, 18, or 20°C and 15 μm m^−2^ s^−1^ with a light: dark cycle of 12:12 h dark cycle. All strains were harvested during the exponential phase, as previous studies have shown that the ploidy levels are often higher during this phase than in the linear or stationary phase (Griese et al., 2011). For DNA extraction, each culture was harvested and centrifuged at 8 min at 8000*g*. Simultaneously, samples for cell enumeration by flow cytometry were preserved in glutaraldehyde (0.25% final concentration). The cell pellet and samples for flow cytometry were stored at −80°C until further analysis.

### Primer design

Commonly used rbcL primers for marine and freshwater strains (Doron et al., 2016; Griese et al., 2011) did not produce amplificons for the brackish strains. To design new primers, rbcL sequences were obtained from whole genome sequence data from strains KAC102, KAC105, KAC106, KAC108, and KAC114. The software MEGA X1 was used to visualize and align the sequences. The rbcL gene was highly conserved and degenerate primers (KAC_rbcLf: CGCGAYCGBATGAACAAGTAY, KAC_rbcLr: CGTCGTCYTTRGTGAAGTCSAG) were designed to target all strains. Additionally, primers from the literature (Syn_rbcL_f: TTCATCAAGAGCTGCTACGG, Syn_rbcr: GACGGCCGTACTTGTTCATC) (Doron et al., 2016) were used for the marine reference strains. All primers had an annealing temperature of 60°C and amplicon sizes between 100 and 200 bp.

**TABLE 1:** Overview of picocyanobacteria with experimentally determined ploidy levels.

Strain	Genome copy number per cell	Environment	Reference
*Synechococcus* PCC 6301	2 to 6	Freshwater	Binder & Chisholm, 1990
*Synechococcus* PCC 7942	3 to 5	Freshwater	Griese et al., 2011; Mori et al., 1996
*Synechocystis* PCC 6803 (substrain “motile”)	58 to 218	Freshwater	Griese et al., 2011
*Synechocystis* PCC 6803 (substrain GT)	43 to142	Freshwater	Griese et al., 2011
Synechocystis PCC 6803 (kazusa)	4 to 53	Freshwater	Griese et al., 2011; Labarre et al., 1989; Zerulla et al., 2016
*Gleobacter* PCC 7421	4 to 5	Freshwater	Watanabe, 2020
*Synechococcus* WH 7803	2 to 5	Marine	Binder & Chisholm, 1995; Griese et al., 2011; Perez‐Sepulveda et al., 2018; this study
*Synechococcus* WH 8103	1 to 2	Marine	Binder & Chisholm, 1995; Burbage & Binder, 2007; Griese et al., 2011, this study
*Synechococcus* WH 7805	1	Marine	Binder & Chisholm, 1995
*Synechococcus* WH 8101	1	Marine	Armbrust et al., 1989
*Prochlorococcus* CCMP 1375	1	Marine	Watanabe, 2020
*Prochlorococcus* MIT 9312	1	Marine	Burbage & Binder, 2007; Zborowsky & Lindell, 2019
*Synechococcus* WH8109	4	Marine	Zborowsky & Lindell, 2019
*Synechococcus* CC9605	1	Marine	Zborowsky & Lindell, 2019
*Synechococcus* WH8102	2	Marine	Zborowsky & Lindell, 2019
*Synechococcus* CC9311	1	Marine	Zborowsky & Lindell, 2019
*Synechococcus* BL107	4	Marine	Zborowsky & Lindell, 2019
*Synechococcus* CC9902	1	Marine	Zborowsky & Lindell, 2019
*Synechococcus* WH7805	1	Marine	Zborowsky & Lindell, 2019
*Synechococcus* RS9917	1	Marine	Zborowsky & Lindell, 2019
*Synechococcus* RS9916	1	Marine	Zborowsky & Lindell, 2019
*Synechococcus* WH5701	4	Marine	Zborowsky & Lindell, 2019
*Synechococcus* RCC307	1	Marine	Zborowsky & Lindell, 2019
*Synechococcus* MITS9220	5	Marine	Zborowsky & Lindell, 2019
*Prochlorococcus* MED4	1	Marine	Zborowsky & Lindell, 2019
*Prochlorococcus* MIT9515	1	Marine	Zborowsky & Lindell, 2019
*Prochlorococcus* MIT9215	2	Marine	Zborowsky & Lindell, 2019
*Prochlorococcus* MIT0604	2 to 3	Marine	Zborowsky & Lindell, 2019
*Prochlorococcus* MATL2A	1	Marine	Zborowsky & Lindell, 2019
*Prochlorococcus* MIT9313	1	Marine	Zborowsky & Lindell, 2019
*Prochlorococcus* SS120	3	Marine	Zborowsky & Lindell, 2019
KAC100	1	Brackish	This study
KAC101	1	Brackish	This study
KAC102	1	Brackish	This study
KAC103	1	Brackish	This study
KAC104	1	Brackish	This study
KAC105	2	Brackish	This study
KAC106	1	Brackish	This study
KAC107	1	Brackish	This study
KAC108	1	Brackish	This study
KAC109	1 to 2	Brackish	This study
KAC110	2 to 3	Brackish	This study
KAC111	1	Brackish	This study
KAC112	3	Brackish	This study
KAC113	1	Brackish	This study
KAC114	1	Brackish	This study
KAC115	3 to 4	Brackish	This study
KAC116	1	Brackish	This study
KAC125	1	Brackish	This study

### DNA extraction and PCR testing

DNA was extracted from cultured KAC strains along with the two marine reference strains *Synechococcus* WH7803, and *Synechococcus* WH8102. Extraction was performed using the FastDNA™ SPIN Kit for Soil from MP Biomedicals Inc. according to the manufacturer's instructions with the addition of incubation with proteinase‐K (0.02 μg μl^−1^, final concentration) at 55°C for 1 h. Sample concentration was measured using an Invitrogen Qubit 2.0 fluorometer (Thermo Fisher Scientific Inc.). Sample purity was assessed using a Thermo Scientific™ NanoDrop 2000 spectrophotometer (Thermo Fisher Scientific Inc.). PCR reactions were performed to test for correct amplification of all primers for both reference strains and KAC strains. The PCR reaction was prepared using the Thermo Scientific Phusion High‐Fidelity PCR Master Mix according to the manufacturer's instructions with a reaction volume of 25 μl. The PCR was performed on a T100™ Thermal Cycler (BIO RAD, USA) with an initial denaturation at 98°C for 30 s; 30 cycles of denaturation at 98°C for 10 s, annealing at 60°C for 1 minute, and extension at 72°C for 5 s; and a final extension step at 72°C for 2 min. A no‐template control using water was included in all runs to check for contamination.

### Quantitative PCR

A serial dilution was performed using brackish KAC strains and marine reference strains *Synechococcus* WH7803 and WH8102 to test for qPCR efficiency of primer sets KAC_rbcL and Syn strains_rbcL. A range of 1–20 ng of gDNA total input was used per reaction. The average efficiencies for each assay were, 91% for the KAC_rbcL assay and 99% for marine_Syn strains_rbcL assay. All samples used in the experiment were diluted to a final concentration of 10 ng μl^−1^. qPCR reactions were prepared using PowerUp™ SYBR™ Green Master Mix (Thermo Fisher Scientific Inc.). Each sample was run in four replicates using a 2 μl DNA template, 5 μl Master Mix, 0.3 μM of each primer, and UltraPure™ DNase/RNase‐Free Distilled Water (Invitrogen™) to a final reaction volume of 10 μl. Reactions were run on a LightCycler® 480 Instrument (Roche) with the following thermocycling settings, according to the manufacturer's instructions: 50°C for 2 min, 95°C for 2 min, 40 cycles of 95°C for 15 s followed by 60°C for 1 min, and a final step for the melt curve analysis of 95°C for 15 s, 60°C for 1 min and 95°C for 15 s. No template controls, with nuclease‐free water instead of sample, were added to each primer master mix set and, on all plates, to check for contamination. Melt curve analysis showed no unspecific amplification. Genome copy numbers were calculated as described in Griese et al., 2011. Average values and standard deviations were calculated from the 4 performed replicates (Supplementary Table S1). For clarity and comparison to previous studies, we have chosen to present rounded numbers in Table 1.

### Flow cytometry

All samples were run on a CYTOFlex Flow Cytometer (Beckman Coulter Inc.) equipped with a blue laser (80 mW) at 488 nm and a red laser (50 mW) at 638 nm. For each sample, 50 μl was analysed at an average flow rate of 1 μl s^−1^. For the cell characterization, four optical parameters were used at a logarithmic scale: forward scatter as a proxy for cell diameter, PE (585/42 nm, blue laser dependent) as a proxy for phycoerythrin content, PC5.5 (690/50 nm, blue laser dependent) as a proxy for chlorophyll *a*, and APC (660/10 nm, red laser dependent) as a proxy for phycocyanin content.

## RESULTS AND DISCUSSION

This study is the first report of ploidy levels of brackish picocyanobacteria. Similar to previous studies on ploidy in picocyanobacteria (e.g., Griese et al., 2011; Perez‐Sepulveda et al., 2018), we used the *rbcL* gene to quantify the genome copy numbers using qPCR. New primers were developed due to the genomic divergence of brackish strains compared to marine and freshwater counterparts. All tested brackish strains (KAC 100‐116 and KAC 125) had low variation in their genome copy number, containing one to four genome copies (Table 1). The majority (16 out of 18) were haploid or diploid, while strains KAC 115 and KAC112 were oligoploid containing four and three genome copies, respectively (Table 1).

In this study, all experiments were performed at standard growth conditions (details in Aguilera et al., 2023) and the ploidy level was assessed in the exponential growth phase. To ensure that the results were consistent with previous studies, two marine strains, *Synechococcus* strains WH7803 and WH8102, with previously reported ploidy levels, were used as controls (Table 1). As for other studies investigating the ploidy level by qPCR, the genome copy numbers presented in this study represent a population average with a possibility that the copy number of individual cells within a population is variable. Despite being cultivated at the same conditions, the genome copy number varied among our tested brackish strains, illustrating different strategies of ploidy level. Studies suggest that polyploidy can provide an advantage during unfavourable conditions (Lui et al. 2018; Pecoraro et al., 2011; Zerulla et al., 2016). An ecophysiological analysis of five of the brackish strains included in this study showed a large variation among the strains for example in tolerance for high salinity, but more sensitivity towards specific light intensity and temperature optimum. Other picocyanobacterial strains of the KAC collection showed a high tolerance towards low temperatures and lower salinity levels (Aguilera et al., 2023). Intensive work on marine *Synechococcus* isolates also indicates the existence of physiologically specialized ecotypes in closely related lineages (Mackey et al., 2017; Pittera et al., 2014; Sohm et al., 2016). When comparing the physiological diversity of brackish *Synechococcus* strains with their ploidy level, no correlation was found when analysing tolerance to abiotic factors as categorical (Yes/No) variables with Pearson's correlation coefficient test (ploidy/salinity *p* = 0.11; ploidy/temperature *p* = 0.24; ploidy/light tolerance *p* = 0.24), indicating that their physiological boundaries in the environment may not be linked with genome copy number. For an accurate assessment of the effect of environmental conditions on genome copy number in picocyanobacteria, further experiments under variable conditions will be needed.

Ploidy could provide ecological and evolutionary advantages, and the ploidy level can vary with environmental factors and growth phase (reviewed in Anatskaya & Vinogradov, 2022). Multiple genome copies may increase the flexibility in gene expression, which has been observed in a diploid diatom (Mock et al., 2017). Oligoploidy could therefore increase resilience to changing environmental conditions (Makarova et al., 2013; Soppa, 2013). Haploidy may be a trait consistent with living in an oligotrophic environment, where streamlining of genetic processes is advantageous when it comes to resource competition (Perez‐Sepulveda et al., 2018). The brackish strains tested in this study were isolated from the central Baltic Sea, a temperate ecosystem that is highly dynamic (Aguilera et al., 2023; Lagus et al., 2007) and characterized by nitrogen limitation during summer (Alegria Zufia et al., 2021). For marine *Synechococcus* sp. WH7803, nutrient limitation did not affect genome copy numbers as extensively as observed in freshwater *Synechocystis PCC6803* and a freshwater archeon (*Haloferax volcanii*). For instance, changes in phosphate (P) concentrations result in copy number variations from 4 copies in P‐deplete to 35 copies in P‐replete conditions in the freshwater picocyanobacteria strain *PCC 6803*; and 2 (P‐deplete) to 20 (normal growth conditions) in *Haloferax volcanii* (Zerulla et al., 2014, 2016). With 3.7 copies in P‐deplete and 4.8 copies in normal growth conditions, the marine strain *Synechococcus* sp. WH7803 remains more stable (Perez‐Sepulveda et al., 2018). Similar patterns were observed for the genome copy number changes during different growth phases. For example, the marine *Synechococcus* sp. WH7803 showed more stability in ploidy level (3 to 6 copies) compared to freshwater *Synechococcus elongatus* PCC 7942 and PCC 6803 (glucose tolerant wild‐type strain), where genome copy numbers ranged from 2 to 10, and 43 to 142, respectively (Griese et al., 2011; Perez‐Sepulveda et al., 2018; Watanabe et al., 2015).

**FIGURE 1:**
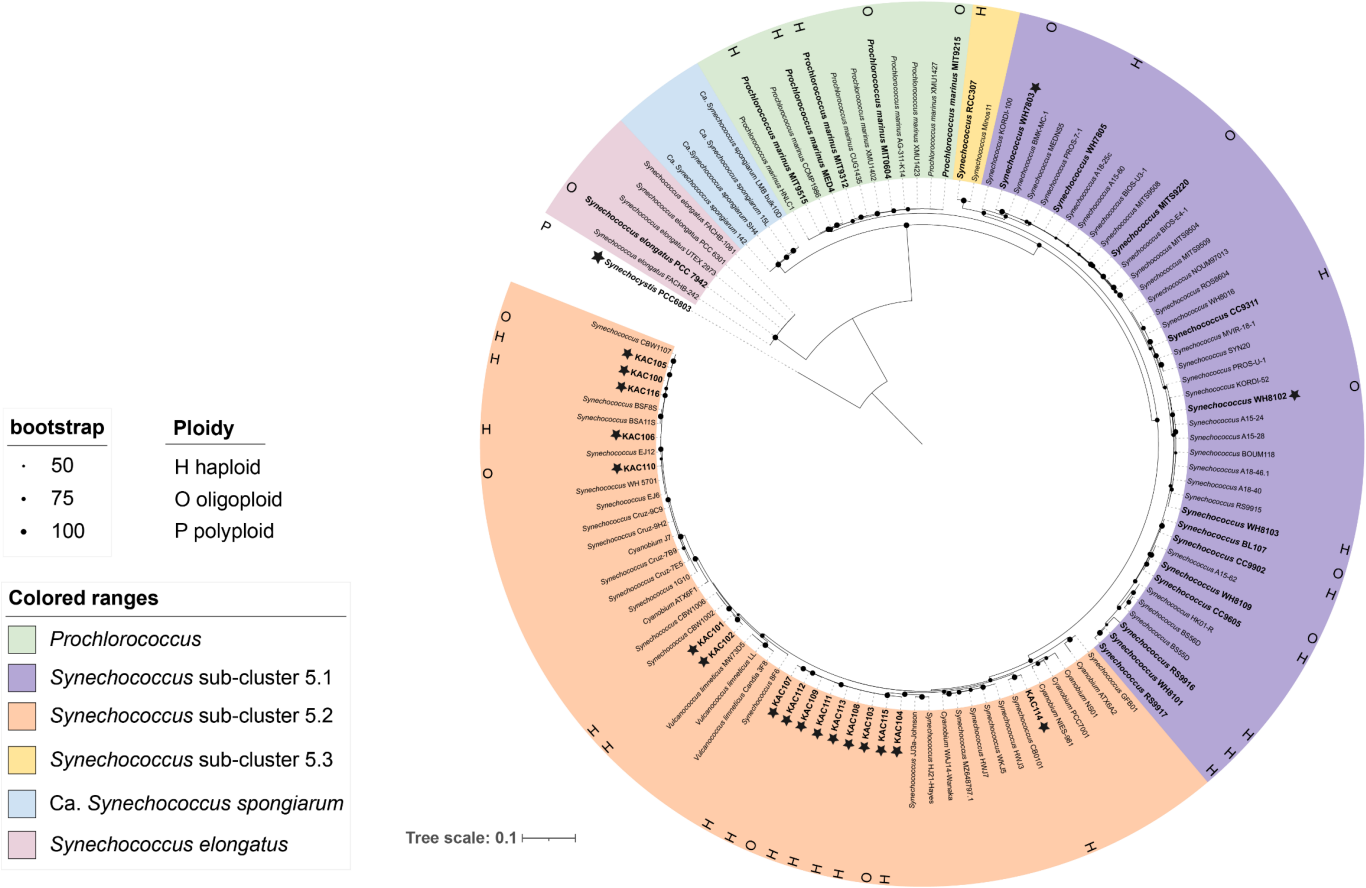
Phylogenetic tree derived from partial 16S rRNA gene sequences using topology given by maximum likelihood (1000 bootstraps). Support values are indicated by the size of internal nodes. Strains with known genome copy numbers from previous studies are indicated in bold and strains with genome copy numbers assigned in this study (brackish strains and two marine strains as control) are additionally marked with a star. Ploidy level is presented by H (haploid), O (oligoploid), or P (polyploid). Designated strains assigned to subcluster 5.3 are based on multigene phylogenies (Cabello‐Yeves et al., 2022; Callieri et al., 2022; Sanchez‐Baracaldo et al., 2019).

Based on 16S rRNA gene phylogeny, the brackish picocyanobacterial strains are assigned to subcluster 5.2 together with some freshwater, estuarine, and halotolerant strains, separated from the marine strains mostly assigned to subcluster 5.1 and 5.3 (Figure 1; Aguilera et al., 2023; Ahlgren & Rocap, 2012). More detailed genomic studies also showed that brackish picocyanobacterial strains contain a mixture of pathways typically found in marine and freshwater species and that they have intermediate genome sizes (Cabello‐Yeves et al., 2022). We therefore hypothesized that brackish strains would have different traits compared to freshwater (oligoploid and polyploid) and marine strains (mostly haploid or oligoploid) when it comes to ploidy level. Available information on ploidy among freshwater strains is limited to *Synechococcus elongatus* PCC 7942 and *Synechocystis* PCC 6803 which do not cluster with the brackish picocyanobacteria strains (Figure 1). Based on the current information on ploidy in freshwater strains, the ploidy level was generally lower among the brackish strains and more similar to the genome copy numbers reported from marine strains (Supplementary Table S1 and Table 1). Among the tested brackish strains, variation in ploidy level was also seen between closely related strains (e.g., 100% 16S rRNA gene identity of KAC 115 [4 genome copies] and KAC 103 [1 genome copy]; Figure 1). This has previously been shown for other prokaryotes including, for example, Gammaproteobacteria (Pecoraro et al., 2011), suggesting that phylogenetic similarity may not select for a certain ploidy level and that phylogenetic analysis itself cannot reveal insights into ploidy level.

It is important to consider ploidy level when interpreting molecular data on relative abundances from environmental samples as polyploidy may lead to an overestimation of certain taxa within the community. In the Baltic Sea, multiple amplicon‐based studies report significant contributions of picocyanobacteria (up to 80%) in 16S rRNA gene amplicon libraries (Andersson et al., 2010; Bertos‐Fortis et al., 2016; Celepli et al., 2017; Lindh et al., 2015). Although the brackish strains generally had low genome copy numbers per cell, in this study, 22% of the brackish strains were at least diploid. However, because of the high similarity of the 16S rRNA gene among co‐occurring brackish picocyanobacterial strains (Aguilera et al., 2023) and the possibility of changing genome copy numbers with different environmental conditions and growth phase (e.g., Ohbayashi et al., 2019; Riaz et al., 2021; Zerulla et al., 2016) integrating a calibration of ploidy level into amplicon sequencing data remains challenging. Further characterization both on the genomic and physiological level (e.g., growth during nutrient depletion and phage susceptibility) will be critical for understanding ploidy strategies among brackish picocyanobacteria as well as for the accurate interpretation of the growing amount of sequencing data.

## AUTHOR CONTRIBUTIONS

**Julia Weissenbach:** Conceptualization; formal analysis; writing – original draft; visualization; writing – review and editing. **Anabella Aguilera:** Writing – review and editing; visualization; formal analysis. **Laura Bas Conn:** Methodology; writing – review and editing. **Jarone Pinhassi:** Writing – review and editing; funding acquisition. **Catherine Legrand:** Writing – review and editing; funding acquisition. **Hanna Farnelid:** Writing – review and editing; resources; supervision; funding acquisition; writing – original draft.

## CONFLICT OF INTEREST STATEMENT

The authors declare no conflict of interest.

## Supporting information


**Table S1:** Supporting Information.

## Data Availability

The data that support the findings of this study are openly available in GenBank at https://www.ncbi.nlm.nih.gov/genbank/, reference number OP441767‐OP441783.
