# Supplementary material for: Ploidy levels in diverse picocyanobacteria from the Baltic Sea
Source: Environ Microbiol Rep. 2024 Sep 17;16(5):e70005. doi: 10.1111/1758-2229.70005 (PMC11405923; doi:10.1111/1758-2229.70005)
Supplement: Supplementary file 1 — Table S1: Supporting Information. [file EMI4-16-e70005-s001.docx]

Supplementary Table S1: **Genome copy number of picocyanobacterial strains determined in this study.** Average values and standard deviations were calculated from the 4 performed replicates.

| Strain | Average genome/ cell ± sd | | |
| --- | --- | --- | --- |
| KAC 100 | 0.97 | ± | 0.01 |
| KAC 101 | 0.76 | ± | 0.14 |
| KAC 102 | 0.95 | ± | 0.26 |
| KAC 103 | 0.86 | ± | 0.01 |
| KAC 104 | 0.89 | ± | 0.01 |
| KAC 105 | 2.29 | ± | 0.12 |
| KAC 106 | 0.65 | ± | 0.10 |
| KAC 107 | 0.87 | ± | 0.07 |
| KAC 108 | 0.88 | ± | 0.14 |
| KAC 109 | 1.46 | ± | 0.08 |
| KAC 110 | 2.26 | ± | 0.22 |
| KAC 111 | 0.94 | ± | 0.03 |
| KAC 112 | 2.78 | ± | 0.09 |
| KAC 113 | 1.19 | ± | 0.05 |
| KAC 114 | 0.84 | ± | 0.06 |
| KAC 115 | 3.63 | ± | 0.19 |
| KAC 116 | 0.88 | ± | 0.02 |
| KAC 125 | 0.52 | ± | 0.12 |
| WH 7803 | 3.84 | ± | 0.19 |
| WH 8102 | 2.05 | ± | 0.06 |
